# ASO Author Reflections: Gut Microbiome Oralization and Intestinal Inflammation After Distal Gastrectomy with Billroth II Reconstruction is Linked to Gastrointestinal Symptoms

**DOI:** 10.1245/s10434-020-08703-3

**Published:** 2020-06-08

**Authors:** Augustinas Bausys, Angela Horvath

**Affiliations:** 1 Department of Abdominal Surgery and Oncology, National Cancer Institute, Vilnius, Lithuania; 2 Clinic of Gastroenterology, Nephrourology and Surgery, Institute of Clinical Medicine, Faculty of Medicine, Vilnius University, Ciurlionio str. 21, 03101 Vilnius, Lithuania; 3 Department of Gastroenterology and Hepatology, Medical University of Graz, Graz, Austria; 4 Center for Biomarker Research in Medicine, Graz, Austria; 5 Department of Transplantation Surgery, Medical University of Graz, Graz, Austria

## Past

Long-term survivors of distal gastric cancer who underwent subtotal gastrectomy with Billroth II reconstruction (SGB2) are likely to experience gastrointestinal symptoms even years after surgery, which impacts negatively on their quality of life.^1^,^2^ The anatomical and physiological changes introduced by SGB2 suppress the gastric acid production and lead to a subsequent increase in gastric pH. This weakens the gastric barrier against food-borne and oral bacteria.^3^

Studies of long-term proton pump inhibitor (PPI) use (i.e. pharmacological suppression of gastric acid) have shown that a breach in gastric barrier is accompanied by very distinct changes of the microbiome that are associated with intestinal inflammation and even mortality.^4^ If similar changes are present after SGB2, this could be relevant for host health and long-term outcome after surgery. Therefore, we investigated if these specific changes are present in the microbiome of patients after SGB2.

## Present

This cross-sectional proof-of-concept study included patients after SGB2 for early gastric cancer, and their non-gastrectomized in-house relatives as controls. Patients showed significant changes in their microbiomes compared with their relatives. These changes bore an obvious resemblance to changes observed in long-term PPI users, including an overrepresentation of *Escherichia-Shigella*, *Enterococcus*, and typical oral bacteria, such as *Streptococcus, Veillonella*, *Oribacterium*, and *Mogibacterium* (i.e. oralization). Furthermore, SGB2 was associated with intestinal inflammation showing an approximately 3.9-fold higher level of fecal calprotectin than their non-gastrectomized relatives. The most commonly documented gastrointestinal symptoms in gastrectomized patients were abdominal discomfort, diarrhea, and bloating, which were associated with distinct taxonomic changes of the gut microbiome.^5^

## Future

This proof-of-concept study provided evidence for gut microbiome oralization and intestinal inflammation after SGB2. Similar to the situation in long-term PPI users, changes in the microbiome of SGB2 patients are associated with gastrointestinal symptoms, such as bloating, diarrhea, or discomfort. If longitudinal studies can confirm the causal link between SGB2 surgery and the here-described novel findings, it could introduce the gut microbiome as a new therapeutic target to improve general host health and quality of life in long-term survivors after SGB2. Microbiome-targeting therapies, such as pro- or prebiotics, fecal microbiota transplant, or even phage therapy, could be trialed to improve patients’ gastrointestinal health and reduce the severe impact of SGB2 on patients’ everyday life and well-being.
